# Multistate model for correlates of parity progression among women living with HIV in Ibadan, Nigeria

**DOI:** 10.21203/rs.3.rs-4927011/v1

**Published:** 2024-09-17

**Authors:** Ahmed Olagunju, Joshua O. Akinyemi, Rotimi Afolabi, Olutosin A. Awolude

**Affiliations:** 1. Department of Epidemiology and Medical Statistics, College of Medicine, University of Ibadan, Ibadan, Nigeria; 2. Department of Obstetrics and Gynaecology, College of Medicine, University of Ibadan/University College Hospital, Ibadan, Nigeria; 3. Infectious Disease Institute, College of Medicine, University of Ibadan, Ibadan, Nigeria; 4. Universidade do Algarve, Faro, Portugal

**Keywords:** Fertility, Parity Progression, Multistate model, Women living with HIV, Fertility rate

## Abstract

Globally, childbearing is a major concern for women living with HIV (WLWH). This study examined parity progression and its predictors among WLWH in Ibadan, Southwest Nigeria. We analysed dataset from a cross-sectional study on childbearing progression among 933 respondents aged 18–49 years receiving care at the HIV Program, University College Hospital, Ibadan. Multistate model was employed for analysis. The adjusted total fertility rate was 3.54. More than 70% were likely to progress from first-to-second birth (HR = 1.77; CI: 1.40, 2.23)but none of the covariates analysed were associated with progression. WLWH were less likely of progression from second-to-third birth (HR = 0.14; 95% CI: 0.13, 0.16). Having 1–2 children at HIV diagnosis (HR = 0.59; CI: 0.48, 0.71), being widowed (HR = 1.36; CI: 1.04, 1.80), having a partner with secondary education(HR = 1.23; 95% CI: 1.02, 1.49), partner’s employment status (HR = 1.40; 95% CI: 1.04, 1.80), , knowledge of partner’s HIV status (Negative) (HR = 0.75; 95% CI: 0.61, 0.94) were associated with progression to third birth. The estimated total fertility rate was lower than the national and the Southwest estimates. Different factors were associated with birth progression from one parity to another.

## Introduction

HIV/AIDS remains a significant global health concern, with approximately 40.1 million lives lost to the disease (1). About 38.4 million people live with HIV at the end of 2021. Additionally, 54% of these people were females (1,2).

About 3.2 million people in Nigeria are living with HIV, making it the country with the second highest burden of the virus globally. The annual estimated rate of new infection is 8 per 10,000 population (3). This is an alarming figure, as it implies that the HIV/AIDS burden in Nigeria is significantly higher than the global average of 0.7% (4). Women are at increased risk of HIV infection due to biological vulnerabilities, epidemiological factors, and the dominant sexual practices of males(5) including HIV being more easily transmitted from men to women than from women to men (6).

Antiretroviral therapy (ART) has been available in Nigeria since the early 2000s, but its acceptance and uptake has varied over time (7). Initially, ART was met with some skepticism and resistance in Nigeria, partly due to concerns about the cost and availability of the drugs, as well as limited knowledge about HIV and AIDS. A review of ART coverage in the countries with the highest burden of HIV revealed that Nigeria had the highest increase in ART coverage by 72% from 2015 – 2020 (8). Data from the World Bank (9) also approximated that ART coverage in Nigeria is 90% in 2021.

Despite the status of HIV among women in sub-Saharan Africa, research have shown that women living with HIV/AIDS (WLWH) desire to have children (10–13). Other researchers have identified age, being married, duration of marriage, high educational status, income, partner’s HIV status, non-use of contraceptives, number of surviving children, remarriage and ART adherence as predictors of fertility desire among HIV patients (12,14,15).

The parity progression among WLWH compounds the increased health risk of women and children, access to care, vertical transmission, etc. Studies on parity progression among WLWH is very important to inform actions that could address these challenges. Researchers have examined fertility desire among WLWH in Nigeria (16–20); however, it is important to explore the parity progression and its predictors. There, this study explored parity progression and its predictors among WLWH in Ibadan, Nigeria over time.

## Methods

### Study Design

The study was cross-sectional in design. It used data collected in a study on childbearing progression and proximate determinants of fertility among WLWH in Ibadan. Details on the design, data collection and study context have been reported in prior publications (15,16,19)

### Study Area

The University College Hospital, Ibadan is a large medical facility with a capacity of 850 beds and is the first tertiary hospital in Nigeria. It is the teaching hospital to the College of Medicine, University of Ibadan. The Antiretroviral Clinic of the facility was established in 2002 as one of 25 Antiretroviral Clinics nationwide, it provides services to the southwestern region of Nigeria and surrounding areas.

### Data Collection Technique

Data was collected from those who had received care for at least one year in the HIV Program located in the Infectious Disease Institute, College of Medicine, University College Hospital/University of Ibadan. The HIV program currently provide care for about 7,000 people living with HIV (PLHIV) and many other seeking HIV prevention services like HIV testing services (HTS), HIV Pre-exposure prophylaxis (PrEP); HIV post-exposure services (PEP).

The questionnaire used to collect information was a validated instrument adapted from the Nigeria Demographic and Health Survey (21) and questions related to HIV care and treatment were adapted from a prior survey on fertility in the era of HIV which was conducted in Nigeria and Zambia (16,22).

### Variables

The main independent variables used for this study were categorized into two subgroups which are: sociodemographic characteristics of women (e.g age at diagnosis, ethnicity, religion, education, employment and number of children at HIV diagnosis), marital profile (marital status at diagnosis, partner’s education, partner’s employment, desire more children and family setting).

The intermediate variables used for this study is HIV Care profile of the women which are ART duration, status disclosure to partner, HIV duration and partner’s HIV status. These variables have been used in previous publication (16).

The dependent variable for this study was progression to the next birth. We obtained this information by extracting the full birth history of the women from the data collected.

### Data Analysis

Descriptive analysis of socio-demographic characteristics of women, marital profile and HIV care profile including parity progression were presented using frequency and percentages; while quantitative variables were described using mean and standard deviations, or median and interquartile range as appropriate. To estimate the total fertility rate, Synthetic Gompertz Relational model was employed (23,24). Multistate model was thereafter used to examine factors associated with parity progression. Firstly, we fitted a model that revealed the baseline hazard of progression to higher order of birth. Secondly, we assessed the effect of independent variables on parity progression among the women at different progressions.

### Multistate Model

A multistate process is a type of random process denoted as X(t), where *t* is a time variable that belongs to a finite time interval *T*=[0, τ]. The process has a finite number of states, S = 1,2, …, N}, in this case, each case represents a different birth event. For example, state 1 is represented as first birth, state 2 is second birth and so on until fifth birth or higher.

At any given time *t*, the process is in one of these states. As the process evolves over time, a history *H*_*t*−_ is generated. This history includes observations of the process over the time interval [0, t), including the states that have been visited previously and the times of transitions between these states. The multistate process can be fully characterized by the probabilities of transitioning between states *h* and *j*.


$$\text{Phj}\left(\text{s},\text{t}\right)=\text{p}\left(\text{X}(\text{t})=\text{j}|\text{X}\left(\text{s}\right)=\text{h},H_{s-}\right)\;for\;h,j\in S;\;s,t\in T;\;s\leq t$$

Where p_hj_(s, t) represents the likelihood of transitioning from state ℎ (e.g., the first birth) at time s to state *j* (e.g., the second birth) at time *t*, given the history up to time *t*.

#### Transition Intensities

Transition intensities α_ℎ*j*_(*t*)describe the propensity of transitioning from state h to state j. It provides an explicit view of the dynamics in transitioning from one birth to another at any given time t.


(1) $$\alpha_{h,j}\left(t\right)=\underset{\Delta t\to 0}{\text{lim}}\frac{p_{h,j}\left(t,t+\Delta t\right)}{\Delta t}$$

#### Model Definition

The multistate process is defined by its transition probabilities between states h and j. The transition probabilities assume to exist, which indicate the immediate risk of moving to state *j* (e.g., from second to third birth) from state *h*.

Considering modeling the birth progression of women from the first to the fifth birth. The states S represent the number of births: S = 1, 2, 3, 4, 5}

At any time t, a woman can be in one of these states. The transition probabilities p_hj_(s, t) might represent the likelihood of progressing from the first birth to the second birth, from the second to the third, and so on, within the time interval [s,t].

The transition intensity α_23_(*t*) could represent the instantaneous risk of moving from the state of having two to having three children at time *t*.

## Results

### Women Characteristics

The background characteristics of the women are shown in Table 1. The mean age of the women was approximately 38.0±6.1 years. Most respondents were aged 35–39 years (29.7%), followed by those aged 40–44 (27.0%). The majority of the women were Yoruba (80%), Christians (61.0%) and currently working (90.4%). Almost half of the women (47%) had completed secondary school education.. At the time of HIV diagnosis, about 48% of the women had between 1–2 children, 35.7% had 3 and more than 3 children and more, while 16.7% did not have a child (Table 1).

More than three-quarter of the women (77.3%) were currently married, Most women partners had secondary education (48.4%), and were employed (84.8%). More than half of the women desired more children (53.6%), while nearly two-thirds were in monogamous relationship (65.6%).

The average HIV duration among the respondents was 6.3±3.4 years. Nearly two-thirds of the women had been living with HIV for 3–8years (62.3%)About 37% of the women had been on ART between 3 – 5 years, while 18.4% had been on ART in less than 2 years. Nearly 80% of the women have disclosed their HIV status to their partner, and half of the respondents’ partner were HIV negative (48.0%), 27.5% were tested positive to HIV while 24.4% of the women did not know their partner’s HIV status (Table 1).

### Total fertility rate estimate

The age-specific fertility and total fertility rates among the studied women were presented in Table 2. Of 933 women, a total of 415 births were recorded 12 months before the survey, and the total children ever born were 2848 children. The corrected total fertility rate for the women was 3.54, the average number of children a woman would have by the end of her childbearing years if she bore children at the current age-specific fertility rates is presented in Table 2.

### Birth progression among women living with HIV

**Fig. 1:**
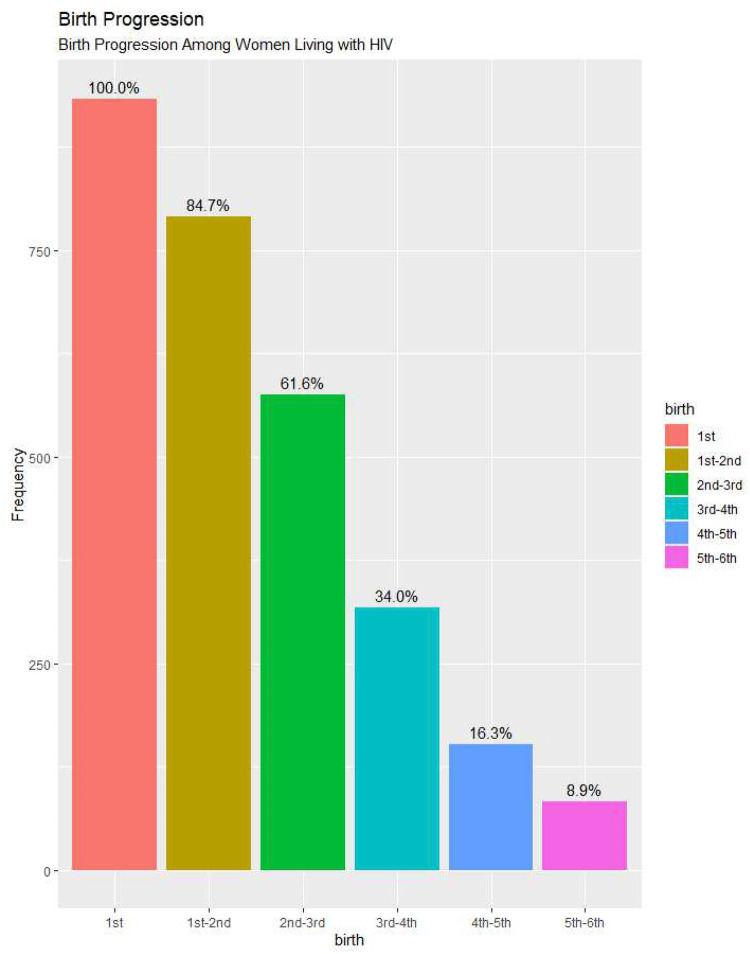
Birth progression among women WLWH

Figure 1 below summarised the percentage of birth progression among 933 respondents covered in this study. Out of the 933 women that had their first, 84.7% of them proceeded to have a second child. The progression from second birth to third birth is 61.6%. Among the people that had third birth, about half of them did not progress to fourth birth, only 34% had fourth child.

There is a sharp decline in the percentage of women that moved from fourth to fifth order and fifth birth to higher births, 16.3% of women moved to fifth birth, while only 8.9% progressed to higher order of birth.

### Birth progression by timing of HIV diagnosis

**Fig. 2:**
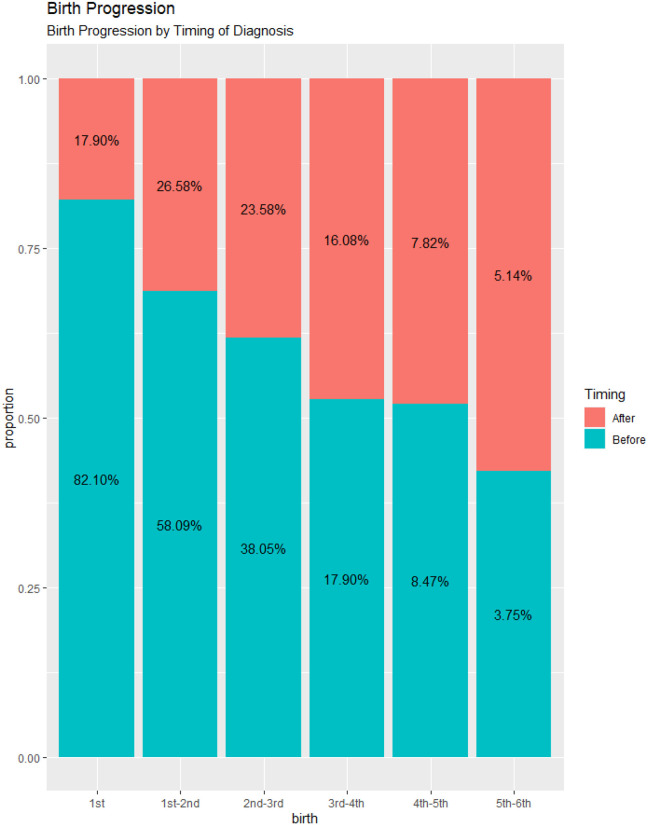
Birth progression by timing of HIV diagnosis

The results showed that about 82% of the women had first birth before HIV diagnosis. Among the women that progressed to second birth, 58% were before diagnosis 27% were after diagnosis. From second to third, 38% had the third child before diagnosis, 24% after diagnosis. At higher order of birth, birth progression before or after diagnosis are almost equal. The proportion of women that progressed to 6th birth after HIV diagnosis are somewhat higher than before diagnosis (Fig. 2).

### Multistate Model for Parity Progression Among WLWH

Table 3 shows the baseline hazard rate of transition from first birth to second birth, second birth to third birth, third birth to fourth birth, fourth birth to fifth birth, and sixth birth. The results showed that women living with HIV are 77% more likely of progressing from their first birth to their second birth (HR = 1.77, 95% CI: 1.40, 2.23). The likelihood of progressing from a second birth to a third birth decreased, women are 76% less likely to progress to third birth after second birth (HR = 0.14, 95% CI: 0.13, 0.16).

The hazard of transition from third birth to fourth birth was 72% less likely among women living with HIV (HR = 0.18, 95% CI: 0.15, 0.20), at fourth birth to fifth birth and fifth birth to sixth birth, the hazards of progression are equal, the rate of transition is 75% less likely among the women (HR = 0.15, 95% CI: 0.13, 0.19)

### Factors associated with parity progression

The results from the Multistate model showed that women have a 77% greater likelihood of progressing from their first birth to their second birth (HR = 1.77, 95% CI: 1.40, 2.23), but none of the factors studied were statistically significant for the progression.

The likelihood of progressing from a second birth to a third birth decreased by 76% (HR = 0.14, 95% CI: 0.13, 0.16), and several factors were found to be statistically significant for this progression, these include the number of children at diagnosis, marital status, educational attainment, employment status of the partner, desire for more children, and partner’s HIV status. Compared to women with women with no formal education, those with primary and tertiary education were less likely of progression to second birth.

Progression from second to third birth was 94% more likely and 41% less likely among women with 3+ (HR = 1.94, 95% CI: 1.61 ,2.40) and 1–2 (HR = 1.34, 95% CI: 1.01, 1.80) children at diagnosis relative to women with no child at diagnosis. Women who were widowed (HR = 1.36, 95% CI: 1.04, 1.80) had 36% increased risks of progression to third birth among the women, though the likelihood of progression was higher among women that have never married compared to the married women. Women whose partners attained secondary education (HR = 1.23, 95% CI: 1.02, 1.49) were 23% more likely to progress to third birth compared with partners with no education. Regarding partner’s employment status, women who had unemployed partners (HR = 1.40, 95% CI: 1.04, 1.80) were 40% more likely to progress to third birth to women whose partners were employed. The results revealed that women whose partner completed only primary education were consistently at increased risk of progressing to higher order of births. Women that desire more children (HR = 0.65 (0.53, 0.78) were about 35% less likely to having third birth after having second birth. Women whose partner has negative status (HR = 0.75, 95% CI: 0.61, 0.94) were 25% less likely to having third birth.

From third birth to fourth birth, the rate of transition were 72% less likely (HR = 0.18, 95% CI: 0.15, 0.20), number of children at diagnosis was statistically significant. Women who had more than two children at diagnosis (HR = 1.34, 95% CI: 1.01, 1.80) were 34% more likely to progress to fourth child.

Transitioning from fifth birth to sixth birth were 75% less likely among the women (HR = 0.15, 95% CI: 0.11, 0.21). Women who completed secondary education (HR = 0.30, 95% CI: 0.10, 0.94) had 70% lesser risks of progression to sixth birth. Similarly, women who had primary or tertiary education had lower likelihood of progression to sixth birth relative to women with no education. Women whose partner had primary and tertiary education were at increased risk of progression to sixth birth relative to women whose partner had no education.

## Discussion

This study employed multistate model to examine parity progression among women receiving HIV care at University College Hospital, Ibadan, Nigeria. The corrected total fertility is low compared to the southwest region estimate of total fertility rate of 4.4 children per women (25).

It was observed from this study that childbearing progression dropped mostly at birth order 3. This also corroborates the women’s desire to use childbirth as a means of coping with HIV diagnosis according to similar studies in Nigeria and Canada (16,26) and it is also a pointer to the importance of integrating reproductive counselling to care of HIV.

This study revealed that women’s level of education was one of the factors associated with parity progression among WLWH. Women having formal education had reduced risk of progressing to higher orders birth relative to women with no formal education, this aligns with other findings in Europe (27,28). This study found that formal education was a protective factor to parity progression at high order of birth.

Women living with HIV were less likely of progressing to higher order of births, progression from second birth to third birth was lower among the women. Among the factors influencing parity progression is number of children at diagnosis, women that had 1–2 children were less likely of progressing to third birth, while women with more than 2 children were more likely of progressing from second to fourth birth. Studies in Nigeria (16,17) found that the higher the number of children at diagnosis, the less is the likelihood of having another child after HIV diagnosis.

It is also worthy to note that women whose partner had formal education were more likely of progressing to second and third and sixth birth. Despite the common assumption that higher education leads to better social and economic status for women and provides them with opportunities outside of childbearing, having an educated partner increased childbirth rates among these women. This may be due to the partner’s influence in reproductive decision-making and their knowledge of the preventative effects of ART on HIV transmission (16,29).

Additionally, desire for more children lowers the risk of progression, women living with HIV may choose to have more children to strengthen their relationship, especially if their partner has not fathered a child yet (12,16,29)

## Limitations

The computation of age-specific fertility rates, total fertility rate, and parity progression relied on the accuracy of the full birth history provided by the respondents. However, there is a possibility of recall bias as some respondents may have forgotten the exact date of birth of their child(ren). The Multistate model used in the study also depended on the accuracy of the birth history, and any inconsistencies were removed using the complete case method. Additionally, the sample size of the study may limit its generalizability to the entire population.

### Conclusions

Exploration of parity progression among women living with HIV provides information on fertility transition, prevention of mother-to-child transmission, maternal and child health and reproductive health of this population. The outcome of this study revealed that women’s educational attainment, number of children at diagnosis, marital status, partner’s educational attainment, partner’s employment status, desire for more children, antiretroviral therapy duration, knowledge of HIV partner’s status were significantly associated with parity progression among women living with HIV in Ibadan, Nigeria.

## Figures and Tables

**Table 1:** Background Characteristics of women living with HIV, Ibadan

Variable	Frequency (n = 933)	Percentage
**Socio-demographic Characteristics**		
**Age group (38.01 ± 6.08)**		
<29 years	75	8.0
30 – 34 years	173	18.5
35 – 39 years	277	29.7
40 – 44 years	252	27.0
45 – 49 years	156	16.7
**Ethnicity**		
Yoruba	751	80.5
Igbo	77	8.3
Others	105	11.3
**Religion**		
Christianity	567	60.8
Islam	366	39.2
**Education**		
No education	63	6.8
Primary	195	20.9
Secondary	443	47.5
Tertiary	232	24.9
**Employment**		
Working	843	90.4
Not working	90	9.6
**Number of Children at HIV Diagnosis**		
None	156	16.7
1 – 2	444	47.6
3+	333	35.7
**Marital Profile**		
**Marital Status**		
Married	721	77.3
Never Married	9	1.0
Divorce/Separated	81	8.7
Widowed	122	13.1
**Partner’s Educational Status (n = 918)**		
None	38	4.1
Primary	99	10.8
Secondary	444	48.4
Tertiary	337	36.7
**Partner’s Employment Status (n = 915)**		
Employed	776	84.8
Unemployed	139	15.2
**Desire more children**		
Not Desired	433	46.4
Desired	500	53.6
**Family Setting (n = 908)**		
Polygamous	312	34.4
Monogamous	596	65.6
**HIV Care Profile**		
**ART Duration (n = 906)**		
<2 years	167	18.4
3 – 5 years	333	36.8
6 – 8 years	250	27.6
9+ years	156	17.2
**Status Disclosure to Partner (n = 919)**		
Disclosed	727	79.1
Non-disclosed	192	20.9
**Partner’s HIV Status**		
Positive	257	27.5
Negative	448	48.0
Unknown	228	24.4
**HIV Duration (6.34 ± 3.38)**		
< 2 years	116	12.4
3 – 5 years	294	31.5
6 – 8 years	287	30.8
9+ years	236	25.3

**Table 2:** Age-Specific Fertility Rate and Total Fertility Rate

		Corrected ASFRs			
Ages	Average Parities	(shifted age)	(true age)	Parities	P/F Ratios
**10–14**			0.057	0.128	
**15–19**	1	1.000	0.195	0.840	0.4975
**20–24**	1.5	1.000	0.151	1.671	0.1985
**25–29**	2.4407	0.780	0.112	2.311	0.1995
**30–34**	2.5896	0.647	0.086	2.801	0.1653
**35–39**	3.0614	0.736	0.066	3.179	0.1702
**40–44**	3.2421	0.131	0.037	3.442	0.1681
**45–49**	3.6410	0.026	0.007	3.545	0.1857
**Total fertility**				**3.54**	

**Table 3:** Hazard Ratio for likelihood of birth progression among WLWH in Ibadan, Nigeria

Progression	n (%)	HR (95% CI)
1^st^	933(32.7)	
1^st^ – 2^nd^ Birth	790(27.7)	**1.77 (1.40, 2.23)**
2^nd^ – 3^rd^ Birth	575(20.2)	**0.14 (0.13, 0.16)**
3^rd^ – 4^th^ Birth	317(11.1)	**0.18 (0.15, 0.20)**
4^th^ – 5^th^ Birth	152(5.3)	**0.15 (0.13, 0.19)**
5^th^ – 6^th^ Birth	83(2.9)	**0.15 (0.11, 0.21)**

HR = Hazard Ratio; CI = Confidence Interval; Bold face values indicate significance

**Table 4:** Multistate Model of Factors Associated with Parity Progression among WLWH

	1^st^ – 2^nd^ HR (95% CI)	2^nd^ – 3^rd^ HR (95% CI)	3^rd^ – 4^th^ HR (95% CI)	4^th^ – 5^th^ HR (95% CI)	5^th^ – 6^th^ HR (95% CI)
**Education**					
None (Ref. Cat.)	1	1	1	1	1
Primary	0.89 (0.29, 2.40)	1.09 (0.73, 1.60)	0.90 (0.52,1.60)	0.82 (0.38, 1.70)	0.51 (0.18, 1.40)
Secondary	1.04 (0.35, 3.09)	0.96 (0.66, 1.40)	0.77 (0.45, 1.31)	0.56 (0.27, 1.19)	**0.30 (0.10, 0.94)**
Tertiary	0.99 (0.34, 2.90)	0.84 (0.56, 1.30)	0.66 (0.36, 1.20)	0.51 (0.21, 1.20)	0.65 (0.19, 2.20)
**Number of Children at HIV Diagnosis**					
None (Ref. Cat.)	1	1	1	1	1
1 – 2	1.13 (0.70, 1.81)	**0.59 (0.48, 0.71)**	0.78 (0.58,1.05)	1.03 (0.66, 1.62)	1.11 (0.52, 2.40)
3+	0.73 (0.46, 1.20)	**1.94 (1.61 ,2.40)**	**1.34 (1.01, 1.80)**	0.97 (0.63, 1.50)	0.90 (0.43, 1.90)
**Marital Status**					
Married (Ref. Cat.)	1	1	1	1	1
Never Married	1.33 (0.08, 22.10)	4.16 (0.86, 20.20)	1.56 (0.28, 8.70)	0.67 (0.08, 5.50)	2.37 (0.23, 24.80)
Divorce/Separated	0.87 (0.38, 2.00)	0.94 (0.65, 1.30)	0.72 (0.41, 1.30)	0.86 (0.33, 2.30)	1.89 (0.51, 7.00)
Widowed	0.99 (0.54,1.80)	**1.36 (1.04, 1.80)**	1.14 (0.79, 1.60)	1.05 (0.64,1.70)	0.79 (0.32, 2.00)
**Partner’s Educational Status**					
None	1	1	1	1	1
Primary	0.83 (0.42, 1.60)	1.02 (0.76, 1.40)	1.27 (0.85, 1.90)	1.38 (0.80, 2.40)	1.66 (0.73, 3.80)
Secondary	1.01 (0.64, 1.60)	**1.23 (1.02, 1.49)**	0.93 (0.71, 1.23)	0.96 (0.63, 1.45)	0.48 (0.24, 0.98)
Tertiary	1.11 (0.68, 1.82)	0.80 (0.65, 0.98)	0.98 (0.72,1.33)	0.82 (0.50, 1.33)	1.41 (0.65, 3.10)
**Partner’s Employment Status**					
Employed	1	1	1	1	1
Unemployed	1.10 (0.58, 1.90)	**1.40 (1.04, 1.80)**	1.10 (0.77, 1.60)	1.30 (0.78, 2.10)	1.00 (0.46,2.30)
**Desire more children**					
Not Desired	1	1	1	1	1
Desired	1.43 (0.87, 2.35)	**0.65 (0.53, 0.78)**	0.80 (0.59, 1.08)	0.78 (0.48, 1.27)	1.27 (0.57, 2.79)
**ART Duration**					
< 2 years	1	1	1	1	1
3 – 5 years	0.87 (0.54, 1.40)	1.00 (0.82, 1.20)	0.88 (0.65, 1.20)	0.90 (0.58, 1.40)	0.88 (0.41, 1.90)
6 – 8 years	0.99 (0.60, 1.60)	1.02 (0.82, 1.30)	0.95 (0.70, 1.30)	0.97 (0.61,1.50)	1.02 (0.47, 2.20)
9+ years	0.87 (0.54, 1.40)	1.00 (0.82,1.20)	0.88 (0.65, 1.20)	0.90 (0.58, 1.40)	0.88 (0.41,1.90)
**Partner’s HIV Status**					
Positive	1	1	1	1	1
Negative	1.16 (0.69, 1.95)	**0.75 (0.61, 0.94)**	1.07 (0.78, 1.46)	0.87 (0.54, 1.39)	1.49 (0.67, 3.28)
Unknown	1.12 (0.61,2.10)	0.87 (0.67,1.10)	1.07 (0.73,1.60)	1.14 (0.67,2.00)	0.84 (0.33,2.10)

HR – Hazard Ratio, Ref. Cat: Reference Category; Bold face values indicate significance

## Data Availability

The dataset used for the study is not available for public sharing to protect the privacy of the participants.

## List of Abbreviations

AIDS: Acquired Immune Deficiency Syndrome
ART: Antiretroviral Therapy
ASFR: Age specific fertility rate
HAART: Highly active antiretroviral therapy
HIV: Human immune deficiency syndrome
NAIIS: National HIV/AIDS Indicator Survey
WLWH: Women living with HIV
